# Incidence and causative agent distribution of viral-induced paediatric asthma exacerbations under strict infection control measures: a single-centre retrospective study in Japan

**DOI:** 10.1186/s12890-023-02779-9

**Published:** 2023-11-29

**Authors:** Tsuyoshi Sasada, Ken Hayashi, Ikuo Okafuji, Chisato Miyakoshi, Satoru Tsuruta

**Affiliations:** 1 Department of Respiratory Medicine, Kobe City Medical Center General Hospital, 1-1, Minatojima-Minamimachi 2-Chome, Chuo-Ku, Kobe, 650-0047 Japan; 2 Department of Pediatrics, Kobe City Medical Center General Hospital, 1-1, Minatojima-Minamimachi 2-Chome, Chuo-Ku, Kobe, 650-0047 Japan

**Keywords:** Asthma exacerbations, Children, COVID-19, Rhinovirus, Enterovirus, Respiratory syncytial virus, Viral respiratory infections

## Abstract

**Background:**

The prevalence of respiratory viruses in children changed under strict infection control measures during the coronavirus disease 2019 (COVID-19) outbreak. In this study, we investigated the frequency of viral detection in the nasopharynx of paediatric patients with asthma exacerbations requiring hospitalization during the COVID-19 pandemic, as well as the distribution of causative viruses.

**Methods:**

We included paediatric patients admitted for asthma exacerbations between November 2020 and December 2022 at a single centre in Kobe, Japan. Demographic, clinical, and laboratory data were collected from their medical records and using additional questionnaires. All patients enrolled in this study met the diagnostic criteria for asthma exacerbations outlined in the Japanese Pediatric Guideline for the Treatment and Management of Bronchial Asthma 2020. Statistical differences were calculated using univariate analyses (chi-square or Mann‒Whitney U test).

**Results:**

We enrolled 203 children hospitalized for asthma attacks and collected nasopharyngeal samples from 189 patients. The median patient age was 3.0 years. Asthma severity was classified as mild (4.0%), moderate (82.3%), or severe (13.8%). The proportion of viral respiratory infections was 95.2% (180/189). The rate of patients with multiple viral infections was 20.6% (39/189). The most frequently detected pathogens were rhinovirus and enterovirus (RV/EV) at 69.3% (131/189), allowing for duplicate detection, followed by respiratory syncytial virus (RSV) at 28.6% (54/189). We also detected RV/EV almost every month compared to RSV and other viruses. In addition, RV/EV-positive patients were significantly older (*p* = 0.033), exhibited higher WBC counts (*p* < 0.001) and higher Eos counts (*p* < 0.001), had elevated total IgE levels (*p* < 0.001) and house dust mite-specific IgE levels (*p* = 0.019), had a shorter duration of hospitalization (*p* < 0.001), and had a shorter duration of oxygen therapy (*p* < 0.001). In patients positive for RV/EV, the use of ICSs significantly reduced the severity of the condition (*p* < 0.001).

**Conclusion:**

Even under strict infection control measures, respiratory viruses were detected in the nasopharynx of almost all paediatric patients who had asthma exacerbations requiring hospitalization.

**Supplementary Information:**

The online version contains supplementary material available at 10.1186/s12890-023-02779-9.

## Introduction

Paediatric asthma exacerbations are closely related to viral respiratory infections [1, 2]. Furthermore, rhinovirus (RV) and respiratory syncytial virus (RSV) infections in infancy increase the risk of asthma at school age [3, 4]. RV is also a major causative pathogen of asthma exacerbations in children [5, 6]. After March 2020, due to the coronavirus disease 2019 (COVID-19) pandemic, normal life changed in terms of social distancing, mask wearing, and hand washing. These changes affected the incidence of viral respiratory infections. A single-centre analysis in Belgium reported that the number of viral respiratory infections decreased from April 2020 to April 2021 [7]. A recent retrospective ecological study in a UK hospital confirmed that the number of children who visited the hospital with a chief complaint of wheezing decreased compared with before and during the COVID-19 outbreak [8]. A meta-analysis revealed a decrease in the incidence of paediatric asthma exacerbations from December 2019 to June 2021 [9]. Some studies also indicated an increase in the proportion of children with well‐controlled asthma [10, 11].

While the prevalence of respiratory viruses declined, that of RV, the most common cause of bronchial asthma exacerbations, increased during the COVID-19 pandemic. Several studies have demonstrated that, compared with that of other seasonal respiratory viruses, the proportion of RV among the causative agents of respiratory infections increased worldwide, including in Japan, after schools reopened in September 2020 [12–16]. Nonetheless, the influence of viruses other than RV cannot be ignored. Therefore, it is difficult to predict the extent to which viral infections affect the incidence of childhood bronchial asthma attacks, particularly over multiple years.

Our study aimed to determine the proportion of asthma exacerbations caused by viral infections during the COVID-19 outbreak and identify the causative viruses.

## Materials and methods

### Study design and population

This study was conducted at Kobe City Medical Center General Hospital, Japan, between November 2020 and December 2022. Our institution is a tertiary care centre equipped with 768 beds. We attended to 18,330 emergency patients in 2020, 21,230 in 2021, and 26,848 in 2022. Throughout the study period, residents of Kobe were advised to adhere to preventive measures, including wearing masks, practising hand hygiene, avoiding indoor gatherings, and self-isolating if exhibiting fever or upper respiratory symptoms. In educational settings such as elementary and nursery schools, both students and staff were mandated to monitor their body temperature and overall health before attendance, ensure regular handwashing, maintain adequate classroom ventilation, and minimize verbal interactions during meals. No lockdowns or major school closures were experienced during this period. Additionally, the vaccination campaign for the adult population was initiated in April 2021 [17]. We retrospectively reviewed the medical records of children hospitalized due to asthma exacerbations. We included only children who met the diagnostic criteria for asthma exacerbations outlined in the Japanese Pediatric Guideline for the Treatment and Management of Bronchial Asthma 2020 (JPGL 2020) [18], which include repetitive typical wheezing, prolonged or chronic cough, medical or family history of allergic diseases, symptoms of airway hyperresponsiveness, breathing style, response to inhalation with short-acting beta-agonists (SABAs), blood and skin test results, and assessment of airway obstruction and inflammation by respiratory tests. Children who met the above criteria but were transferred to other hospitals were also included in the analysis.

### Procedures and definitions

Based on the JPGL 2020 criteria, asthma control was categorized as well-controlled, partially controlled, or uncontrolled [18]. This classification took into account factors such as minor respiratory symptoms, observed acute exacerbations, limitations in daily activities, and the patient's history of SABA use over the preceding month. Patients were considered to have well-controlled asthma if they did not present with any of these factors within the last month. A partially controlled status was ascribed to patients who demonstrated any one of the following within the same timeframe: minor respiratory symptoms occurring at least once a month, occasional limitations in daily activities, or a record of monthly SABA intake. Conversely, patients were labelled as having uncontrolled asthma if they met any of the following criteria within the previous month: minor respiratory symptoms appearing at least weekly, at least one evident acute exacerbation, limitations in daily activities occurring at least once, or the utilization of SABAs at least once per week.

The severity of asthma exacerbations was determined according to the JPGL 2020 [18]. Attacks were diagnosed as mild or moderate when children were calm at rest and had slight wheezing or trapped breathing, with the borderline between the two grades being a peripheral arterial oxygen saturation (SpO_2_) cut-off level of 95% (mild, ≥ 95%; moderate, < 95%). Attacks were considered to be severe if children were restless even at rest and had audible wheezing and visible retractive breathing and an SpO2 < 91%.

The main treatment for paediatric asthma exacerbations was short-acting beta-agonist inhalation and oxygen administration as needed, with systemic steroid and aminophylline infusion in refractory cases. Patients were admitted to the hospital if they had severe exacerbations, did not respond to outpatient treatment within 2 h, or if complications were observed. Patients were discharged when oxygen demand ceased and expiratory effort disappeared during nighttime sleep. The criteria for the duration of hospitalization and oxygen and systemic steroid administration were set according to the JPGL 2020 [18]. The duration of oxygen administration was based on an SpO_2_ level of ≥ 95%, while the duration of systemic steroid administration was determined at the discretion of the attending physician, considering the patient’s response to treatment, the current intensity of long-term treatment, and whether the patient had been hospitalized for an acute exacerbation in the past year.

Blood tests were performed on admission, although not for all patients, and included white blood cell (WBC) and eosinophil (Eos) counts and total and house dust mite-specific immunoglobulin E (IgE) levels. Calculations were performed only for patients who underwent testing.

### Virus detection

Nasopharyngeal samples were analysed using the BioFire® Respiratory Panel 2.1, which can detect major causative respiratory microorganisms [19, 20], including severe acute respiratory syndrome coronavirus 2 (SARS-CoV-2), adenovirus (AdV), coronavirus (HCoV) HKU1/NL63/OC43/229E, parainfluenza virus (PIV) 1/2/3/4, influenza virus (Flu) A/B, RSV, RV/enterovirus (RV/EV), human metapneumovirus (hMPV), *Bordetella pertussis*, *Bordetella parapertussis*, *Chlamydia pneumoniae*, and *Mycoplasma pneumoniae*. This method cannot distinguish between RV and EV because they have genetically similar structures [21, 22].

### Data collection

Demographic (age and sex), clinical (asthma control, controller medications, asthma exacerbation severity, duration of hospitalization, and duration of oxygen and systemic steroid administration), and laboratory data (WBC and Eos counts, total IgE and house dust mite-specific IgE levels, and nasopharyngeal swab test results) were collected from the patients’ medical records. In cases of missing data, we referred to the patients’ parents and administered additional questionnaires.

### Statistical analysis

Collected data were analysed using univariate analyses by employing the chi-square or Mann‒Whitney U test, as appropriate. House dust mite-specific IgE levels below the limit of detection (< 0.1 UA/mL) were regarded as 0.01 UA/mL. JMP® 17 (SAS Institute, Cary, NC, USA) was used for statistical analysis. A p value less than 0.05 was considered statistically significant.

## Results

### Patient characteristics

During the study period, 230 children were hospitalized for the treatment of asthma exacerbations. After excluding 27 children who did not meet the JPGL 2020 criteria, 203 children were included in this study, including three children who were transferred to other hospitals. The patients’ characteristics are presented in Table 1.

**Table 1:** Patient characteristics

Features	Total (*N* = 203)	RV/EV-positive (*N* = 131)	RV/EV-negative (*N* = 58)	*p* value (positive vs. negative)
Age, years, median (IQR)	3.0 (1.8–4.8)	3.2 (1.8–4.8)	2.6 (1.6–3.5)	0.033
Male sex, N (%male)	110 (54.2)	76 (58.0)	26 (44.8)	0.093
Asthma control				0.66
Well-controlled, N (%)	59 (29.1)	41 (31.3)	16 (27.6)	
Partially controlled, N (%)	72 (35.5)	49 (37.4)	20 (34.5)	
Uncontrolled, N (%)	72 (35.5)	41 (31.3)	22 (37.9)	
Controller medications				0.25
LTRAs, N (%)	44 (21.7)	31 (23.7)	10 (17.2)	
ICSs, N (%)	4 (2.0)	3 (2.3)	0	
LTRAs + ICSs, N (%)	30 (14.8)	19 (14.5)	10 (17.2)	
ICSs/LABAs, N (%)	1 (0.5)	0	1 (1.7)	
LTRAs + ICSs/LABAs, N (%)	12 (5.9)	6 (4.6)	6 (10.3)	
LTRAs + ICSs/LABAs + LAMAs + dupilumab, N (%)	1 (0.5)	0	0	
None, N (%)	111 (54.7)	72 (55.0)	31 (53.4)	
White blood cells/μL, median (IQR)	11,450 (8,700–16,125)	13,850 (10,400–17,475)	7,900 (6,300–11,750)	< 0.001
Eosinophils/μL, median (IQR)	71 (0–251)	152 (39–299)	0 (0–39)	< 0.001
Total IgE, IU/mL, median (IQR)	250 (68–808)	334 (129–1,107)	92 (16–390)	< 0.001
House dust mite-specific IgE, UA/mL, median (IQR)	1.9 (0.01–52.6)	8.6 (0.01–68.5)	0.1 (0.01–16.1)	0.019
Duration of hospitalization, days, median (IQR)	5 (4–6)	4 (4–5)	5 (5–7)	< 0.001
Duration of oxygen therapy, days, median (IQR)	2 (1–3)	2 (1–3)	3 (2–5)	< 0.001
Duration of systemic glucocorticoid therapy, days, median (IQR)	3 (2–5)	3 (3–4)	4 (2–5)	0.42
Exacerbation severity				0.62
Mild, N (%)	8 (4.0)	4 (3.1)	3 (5.1)	
Moderate, N (%)	167 (82.3)	111 (84.7)	46 (79.3)	
Severe, N (%)	28 (13.8)	16 (12.2)	9 (15.5)	

*IQR* Interquartile range, *RV* Rhinovirus, *EV* Enterovirus, *LTRAs* Leukotriene receptor antagonists, *ICSs* Inhaled corticosteroids, *LABAs* Long-acting beta-agonists, *LAMAs* Long-acting muscarinic antagonists

*p* value calculated using the Mann‒Whitney U test or the chi-square test

There were 110 (54.2%) boys and 93 (45.8%) girls with a median age of 3.0 years (interquartile range [IQR], 1.8–4.8 years). Asthma control classifications indicated that 59 patients had well-controlled asthma, 72 had partially controlled asthma, and another 72 exhibited uncontrolled asthma. In terms of long-term treatment, 92 patients were on controller medications, while 111 patients were not receiving any such treatments. Of these participants, 44 were treated with leukotriene receptor antagonist (LTRA) monotherapy, 4 received inhaled corticosteroid (ICS) monotherapy, 30 were on a combined regimen of LTRAs and ICSs, 1 was administered inhaled corticosteroid and long-acting beta-agonist (ICS/LABA) combination therapy, 12 received both LTRAs and ICSs/LABAs, and 1 patient was on a comprehensive treatment comprising LTRAs, ICSs/LABAs, long-acting muscarinic antagonists (LAMAs), and dupilumab. The median duration of hospitalization was 5 days (IQR, 4–6 days). Asthma exacerbations were classified as mild in 8 (4.0%), moderate in 167 (82.3%), and severe in 28 (13.8%) patients. Data on WBC and Eos counts and total and house dust mite-specific IgE levels were available for 157/203, 148/203, 131/203, and 129/203 patients, respectively.

### Distribution of detected viruses by infection type

**Fig. 1:**
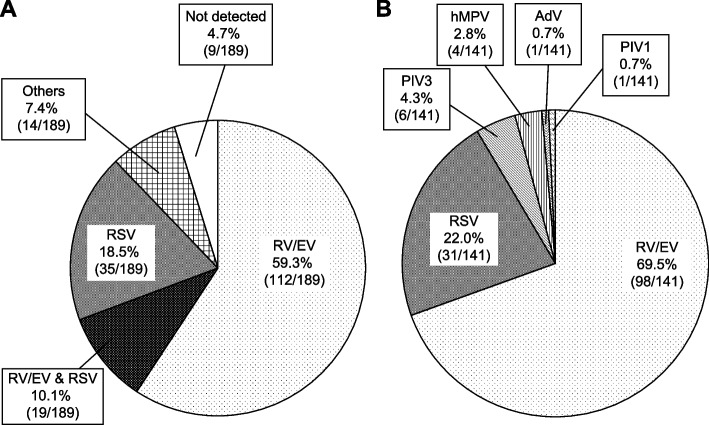
Distribution of viruses detected in the nasopharynx of patients hospitalized due to acute exacerbations of childhood asthma. **A** Distribution in all patients (including duplicate detections); **B** Distribution in patients with single viral infections. RV/EV, rhinovirus/enterovirus; RSV, respiratory syncytial virus; PIV, parainfluenza virus; AdV, adenovirus; hMPV, human metapneumovirus

Nasopharyngeal samples were collected from 189 patients. Eleven patients were not tested in our hospital because they had already been tested in other hospitals. Patients were first assessed at an outpatient clinic before their transfer to our institution. Among them, seven tested negative for COVID-19, three tested positive, and one was not retested owing to a documented recent COVID-19 infection. Three patients underwent only COVID-19 antigen tests, and all tested negative for the virus. The proportion of viral respiratory infections was 95.2% (180/189). The rate of patients with multiple viral infections was 20.6% (39/189). Twenty-seven patients were infected with two types of viruses, eleven patients with three types, and one patient with four types. Among all patients (*n* = 189), the most common causative agents were RV/EV and RSV, accounting for 69.3% and 28.6% of cases, respectively, with coinfection confirmed in 19 (10.1%) patients (Fig. 1A). Among patients with a single viral infection (*n* = 141), RV/EV and RSV were also most commonly confirmed, accounting for 69.5% and 22.0% of cases, respectively, followed by PIV3, hMPV, PIV1, and AdV (Fig. 1B).

### Distribution of detected viruses by period

**Fig. 2:**
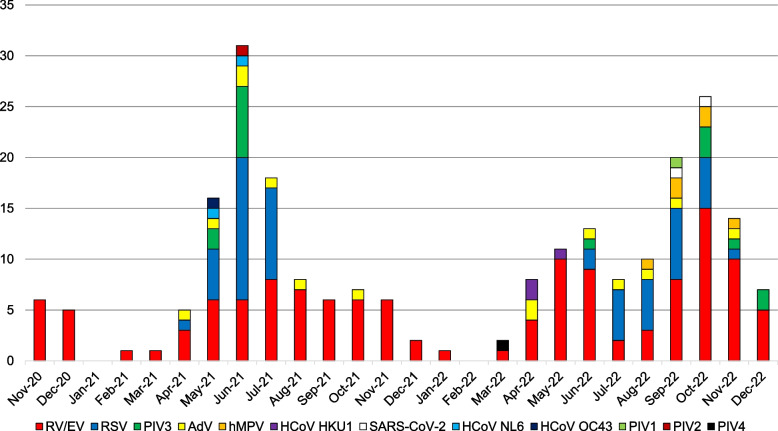
Distribution of detected viruses by month. RV/EV, rhinovirus/enterovirus; RSV, respiratory syncytial virus; PIV, parainfluenza virus; AdV, adenovirus; hMPV, human metapneumovirus; HCoV, coronavirus; SARS-CoV-2, severe acute respiratory syndrome coronavirus 2

The distribution of detected viruses by period is shown in Fig. 2. RV/EV was detected almost every month, whereas RSV, PIV3, and AdV showed significant peaks in the summer months of 2021 and 2022. hMPV was most often detected in the autumn of 2022. Notably, SARS-CoV-2 was detected in only two cases.

### Comparison between RV/EV-positive and RV/EV-negative patients

As RV/EV were the most prevalent causative agents in our study population, we compared the patients’ characteristics according to RV/EV positivity status (Table 1). Patients positive for RV/EV were significantly older (*p* = 0.033), exhibited higher WBC counts (*p* < 0.001) and higher Eos counts (*p* < 0.001), had elevated total IgE levels (*p* < 0.001) and house dust mite-specific IgE levels (*p* = 0.019), had a shorter duration of hospitalization (*p* < 0.001), and had a shorter duration of oxygen therapy (*p* < 0.001). There were no significant differences in sex (*p* = 0.093), asthma control (*p* = 0.66), controller medications (*p* = 0.25), duration of systemic glucocorticoid treatment (*p* = 0.42), or exacerbation severity (*p* = 0.62). In patients who tested positive for RV/EV, the use of ICSs significantly reduced the severity of the condition (*p* < 0.001) (Table 2). Conversely, in RV/EV-negative patients, the severity remained unchanged with ICS use (*p* = 0.51) (Table 2). Furthermore, when comparing those treated with LTRAs to those who were not treated with LTRAs, there was no difference in severity between RV/EV-positive (*p* = 0.39) and RV/EV-negative patients (*p* = 0.74) (Table 2).

**Table 2:** Relationship between control medications and exacerbation severity

(A) LTRA use and exacerbation severity for RV/EV-positive patients			
	LTRA use	No LTRA use	*p* value
Mild, N (%)	3 (2.3)	1 (0.8)	0.39
Moderate, N (%)	47 (35.9)	64 (48.9)	
Severe, N (%)	6 (4.6)	10 (7.6)	
(B) LTRA use and exacerbation severity for RV/EV-negative patients			
	LTRA use	No LTRA use	*p* value
Mild, N (%)	2 (3.5)	1 (1.7)	0.74
Moderate, N (%)	20 (34.5)	26 (44.8)	
Severe, N (%)	4 (6.9)	5 (8.6)	
(C) ICS use and exacerbation severity for RV/EV-positive patients			
	ICS use	No ICS use	*p* value
Mild, N (%)	4 (3.1)	0	< 0.001
Moderate, N (%)	23 (17.6)	88 (67.2)	
Severe, N (%)	1 (0.8)	15 (11.5)	
(D) ICS use and exacerbation severity for RV/EV-negative patients			
	ICS use	No ICS use	*p* value
Mild, N (%)	0	3 (5.2)	0.51
Moderate, N (%)	14 (24.1)	32 (55.2)	
Severe, N (%)	3 (5.2)	6 (10.3)	

*p* value calculated using the chi-square test

## Discussion

In this study, viral respiratory infections were detected in almost all paediatric patients with asthma exacerbations during the COVID-19 pandemic. The proportion of RV/EV and RSV infections was relatively high, which was similar to the trend before the COVID-19 outbreak [23, 24]. Furthermore, the patients with detected RV/EV showed higher Eos counts, total IgE levels, and house dust mite-specific IgE levels.

The characteristics of patients with bronchial asthma did not change before and after the COVID-19 pandemic. In a 2002 study among Japanese school-aged children, the prevalence of bronchial asthma was 8.07% among males and 4.91% among females [25]. A single-centre Japanese study investigating the characteristics of children with asthma exacerbations before the COVID-19 pandemic found that the median WBC count and total IgE level were 12,000/µL (range, 3,400–32,800/µL) and 207 IU/mL (range, 4–11,800 IU/mL), respectively, and the exacerbation attacks were regarded as mild in 21/216 (10%), moderate in 146/216 (68%), and severe in 49/216 (22%) patients [26]. Our results showed the same trend.

Respiratory viral infections have been closely associated with asthma exacerbations in children, even during the COVID-19 outbreak. In this study, viruses were detected in 180 of the 189 (95.2%) tested children, whereas in previous studies, the probability of viral detection was 80–85% [1, 24, 27, 28]. The reason may be that children were less frequently exposed to aggravating factors other than viral infections, such as particulate matter 2.5 (PM2.5), sulfur dioxide, and exercise [29–31]. Both paediatric asthma exacerbations and sulfur dioxide concentrations decreased in Kobe in 2020 during the COVID-19 pandemic [32]. A study reported that air pollution improved in Tokyo because Japan and other Asian countries had reduced economic activity during the lockdown [33].

In this study, RV/EV was most commonly implicated in childhood asthma exacerbations and was detected in 131 of 189 (69.3%) patients. RV was also the most common pathogen in childhood asthma before the COVID-19 outbreak [23, 24, 26]. A meta-analysis that analysed the data on paediatric asthma attacks from 1970 to 2014 confirmed that the rates of asthma attacks among children induced by RV and EV were 45.7% (95% Confidence Interval [CI], 37.5–53.8%) and 11.8% (95% CI, 6.2–22.5%), respectively [24]. Our results are consistent with these previously reported findings.

To the best of our knowledge, no prior study has investigated the change in the rate of RV infections in Japan over 3 years. Our results did not contradict previously reported findings that the number of detected RV infections did not decrease during the COVID-19 outbreak worldwide [14–16]. The present study also showed a cyclical pattern of RV/EV infections throughout the year, which aligns with findings from multiple centres in the United States [34]. However, our study revealed that exacerbations induced by RSV were more common in the summer period. Furthermore, RSV was detected in 28.6% of cases, which is a higher rate than the 17.7% (95% CI, 13.2–23.7%) reported in a meta-analysis before the COVID-19 pandemic [24]. According to sentinel surveillance by the National Institute of Infectious Diseases in Japan, the number of RSV infections in 2021 and 2022 increased compared to that in previous years [35]. The peak of RSV infections was mainly around June or July after the COVID-19 outbreak, whereas it was usually around September before the outbreak [35]. We speculate that this tendency was affected by the reduction in children exposed to RSV as a result of strict infection control. Observational studies have reported the same phenomenon worldwide [36]. Our results reflect these previous findings.

In the present study, the RV/EV-positive group tended to have more Th2 immune reactions than the RV/EV-negative group. Previous reports showed that RV infection was associated with higher serum total IgE, Eos, Eos cationic protein, and interleukin-5 levels than infections with other viruses [37, 38]. ICSs are known to attenuate Th2 immune responses [39]. Our findings, which demonstrate reduced asthma severity in RV/EV-positive patients following ICS administration, further support the link between RV/EV infection and Th2 immune reactions. It was assumed that the biological response to RV did not change significantly even during the COVID-19 pandemic.

Our study had several limitations. First, this was a single-centre study; thus, the results may not reflect the overall trends. Second, the detection of viruses using the BioFire® Respiratory Panel 2.1 also had limitations. Namely, a positive result for Flu, hMPV, PIV, or RSV typically indicates a current infection. Conversely, positive findings for RV/EV or AdV can sometimes reflect past infections as well. Therefore, test results may not always reflect the current infection [40]. Furthermore, there are limitations related to medical examinations. More people tended to refrain from seeking medical care during the COVID-19 pandemic. Therefore, compliance with long-term controlled drugs was likely different from that before the COVID-19 outbreak. It is also possible that children did not seek medical attention even when their asthma attacks were of sufficient severity to warrant hospitalization. A study from Hong Kong observed a decrease in consultations among asthma attack patients aged 18 years and older during the initial phases of the COVID-19 pandemic [41]. These points should be considered when comparing the findings with those from pre-COVID-19 periods. It is difficult to avoid viral exposure for multiple years, even under strict infection control measures, and viral infections are a major cause of childhood asthma exacerbations. Clinicians must maintain compliance with long-term medications and prepare patients for unexpected viral infections.

## Conclusions

The rate of virus-induced asthma exacerbations among children was high even during the COVID-19 pandemic, and RV/EV was the most common cause, similar to before the pandemic. In addition, asthma exacerbations caused by RV/EV were associated with higher Eos, total IgE, and house dust mite-specific IgE levels. Thus, it can be inferred that RV/EV infections are closely related to Th2 cell responses, even during the COVID-19 pandemic.

### Supplementary Information

**Additional file 1:** **Table** **S1.** Comparison between before and after RV/EV infection in White blood cell , Eosinophils , Total IgE , and House dust mite specific IgE.

## Data Availability

The datasets used and/or analysed during the current study are available from the corresponding author upon reasonable request.

## Abbreviations

AdV: Adenovirus
CI: Confidence Interval
COVID-19: Coronavirus disease 2019
Eos: Eosinophil
EV: Enterovirus
Flu: Influenza virus
HCoV: Coronavirus
hMPV: Human metapneumovirus
ICS: Inhaled corticosteroid
IQR: Interquartile range
JPGL 2020: Japanese Pediatric Guideline for the Treatment and Management of Bronchial Asthma 2020
LABA: Long-acting beta-agonist
LAMA: Long-acting muscarinic antagonist
LTRA: Leukotriene receptor antagonist
PIV: Parainfluenza virus
PM2.5: Particulate matter 2.5
RSV: Respiratory syncytial virus
RV: Rhinovirus
SARS-CoV-2: Severe acute respiratory syndrome coronavirus 2
SpO2: Peripheral arterial oxygen saturation
WBCs: White blood cells
