# Supplementary material for: Incidence and causative agent distribution of viral-induced paediatric asthma exacerbations under strict infection control measures: a single-centre retrospective study in Japan
Source: BMC Pulm Med. 2023 Nov 29;23:480. doi: 10.1186/s12890-023-02779-9 (PMC10685531; doi:10.1186/s12890-023-02779-9)
Supplement: Supplementary file 1 — Additional file 1: Table S1. Comparison between before and after RV/EV infection in White blood cell , Eosinophils , Total IgE , and House dust mite specific IgE. [file 12890_2023_2779_MOESM1_ESM.pdf]

Supplementary 1. Comparison between before and after RV/EV infection in White blood cell, Eosinophils, Total IgE, and House dust mite-specific IgE

	White blood cell, /μL		Eosinophils, /μL		Total IgE, IU/mL		House dust mite-specific IgE, UA/mL	
	Before RV/EV infection	After RV/EV infection	Before RV/EV infection	After RV/EV infection	Before RV/EV infection	After RV/EV infection	Before RV/EV infection	After RV/EV infection
Patient1	8,800	7,500	0	75				
Patient2	6,800	11,100	340	611	226	202	29	25.2
Patient3	10,100	9,200	152	46	11	17	1.52	1.84
Patient4	9,700	1,8700	388	94	63		0.01	
